# Combination of Single- and Paired-Pulse Somatosensory Evoked Potentials in Ischemic Monitoring: Preliminary Investigation in Carotid Endarterectomy

**DOI:** 10.7759/cureus.12206

**Published:** 2020-12-21

**Authors:** Hiroshi Fujioka, Eiichirou Urasaki, Yoshiteru Soejima, Hideki Harada, Katsuhiro Yamashita

**Affiliations:** 1 Neurosurgery, Nagasaki Yurino Hospital, Nagasaki, JPN; 2 Neurosurgery, Kanmon Medical Center, National Hospital Organization (NHO), Shimonoseki, JPN; 3 Neurosurgery, Cognitive and Molecular Research Institute of Brain Diseases, Kurume University, Fukuoka, JPN; 4 Neurosurgery, Fukuoka Mirai Hospital, Fukuoka, JPN; 5 Anaesthesiology, Cognitive and Molecular Research Institute of Brain Diseases, Kurume University, Fukuoka, JPN

**Keywords:** carotid endarterectomy (cea), ischemic monitoring, selective neuronal loss, somatosensory evoked potentials (seps)

## Abstract

Introduction

Severe ischemia induces cerebral excitability imbalance before completion of infarct. To investigate the clinical availability of this imbalance with ischemic monitoring, paired-pulse somatosensory evoked potentials (SEPs) were performed in conjunction with conventional SEPs during carotid endarterectomy.

Methods

For carotid endarterectomy patients with hemodynamic deficits of the middle cerebral artery area (n = 34), the excitability imbalances (Q) were measured by paired-pulse SEPs, wherein the second response (A_2_) was divided by the first (A_1_; Q = A_2_/A_1_). Regional cerebral saturation (rSO_2_) was also measured. Occlusion was performed twice using shunting.

Results

Each carotid occlusion induced a significant decrease in mean A_1_ and rSO_2_, and an increase in mean Q values (p < 0.001), which returned to the baseline level after occlusion. While neuronal imbalances were mostly transient, persistently increased Q values were observed in four cases (11.8%), all indicating postoperative abnormalities in diffusion-weighted magnetic resonance imaging (100%). Meanwhile, A_1_ detected the postoperative abnormality in only one case (25%). Preoperative Q values at the time of surgery were significantly higher in symptomatic patients having the upper limb deficits than those without (p < 0.01), indicating persistent or permanent imbalances.

Conclusion

Paired-pulse SEPs reliably identified transient, persistent or permanent neuronal imbalances, depending on the ischemic severity. These preliminary results indicated that paired-pulse SEPs, in combination with conventional SEPs (A_1_), may offer better ischemic monitoring.

## Introduction

Somatosensory evoked potentials (SEPs) can identify cerebral ischemic changes by detecting electric failure in the penumbra, which lies within a narrow hemodynamic window (cerebral blood flow of 12-14 ml/100 g/min). While SEPs are widely used for ischemic monitoring, the unsolved issues of low sensitivity (58%) and false negatives (0 to 3.5%) suggest the need for developing another approach [1].

Growing evidence has indicated that ischemia before completion of infarct, which includes transient ischemic attacks [2, 3], can induce changes in cerebral excitability through the imbalances of excitatory and inhibitory neuronal activities [4, 5]. Further, recent findings on the rescued penumbra or chronic hypoperfusion have demonstrated a salvageable tissue damage, called selective neuronal loss (SNL) [6, 7]. SNL is characterized by a reduction in benzodiazepine receptors in normally appearing cortex [6, 8], which has dynamic properties for its rapid induction (<6 hours from stroke onset in humans [9], and in the order of minutes in animals) [10, 11], and reversibility [10-13].

Although these dynamic properties suggest the potential availability with ischemic monitoring, no previous studies, to our knowledge, have addressed the issue. Cerebral excitability imbalances can be electrophysiologically evaluated by paired-pulse protocols, wherein evoked responses to two successive stimuli, typically within 100 ms in human SEPs, are evaluated [14]. Assuming that paired-pulse SEPs, in conjunction with conventional single-pulse SEPs, could contribute to the better diagnostic accuracy, this study addressed this issue in patients undergoing carotid endarterectomy (CEA).

## Materials and methods

Patients

In this observational study, we analyzed the data of patients with ipsilateral or bilateral carotid artery stenosis who underwent CEA with the following three inclusion criteria: (i) preoperative condition of stage 1 or 2 ischemia upon single photon emission computed tomography (SPECT) in the middle-cerebral artery (MCA) area of the affected hemisphere, and the availability of (ii) intraoperative shunting and (iii) conventional and paired-pulse SEPs (described below). The research was approved by the institutional review board. Informed consent to participate in the study was obtained in written form from each patient.

CEA and the electrophysiological evaluation

Preoperative 123 I-IMP dual-table autoradiography SPECT and magnetic resonance imaging (MRI, 1.5 Tesla) with plaque imaging and carotid duplex were routinely performed. Head computed tomography (CT) was performed before and on the next day after surgery, and diffusion-weighted MRI (DWI) was performed within one week after surgery. Cerebral angiography was performed on high-risk patients.

Occlusion was induced twice during the shunting maneuver (LeMaitre Vascular GmbH, Germany). Anesthesia was maintained by sevoflurane with normocarbia. The monitoring was performed by trained technicians blinded to the objective of the study.

**Figure 1:**
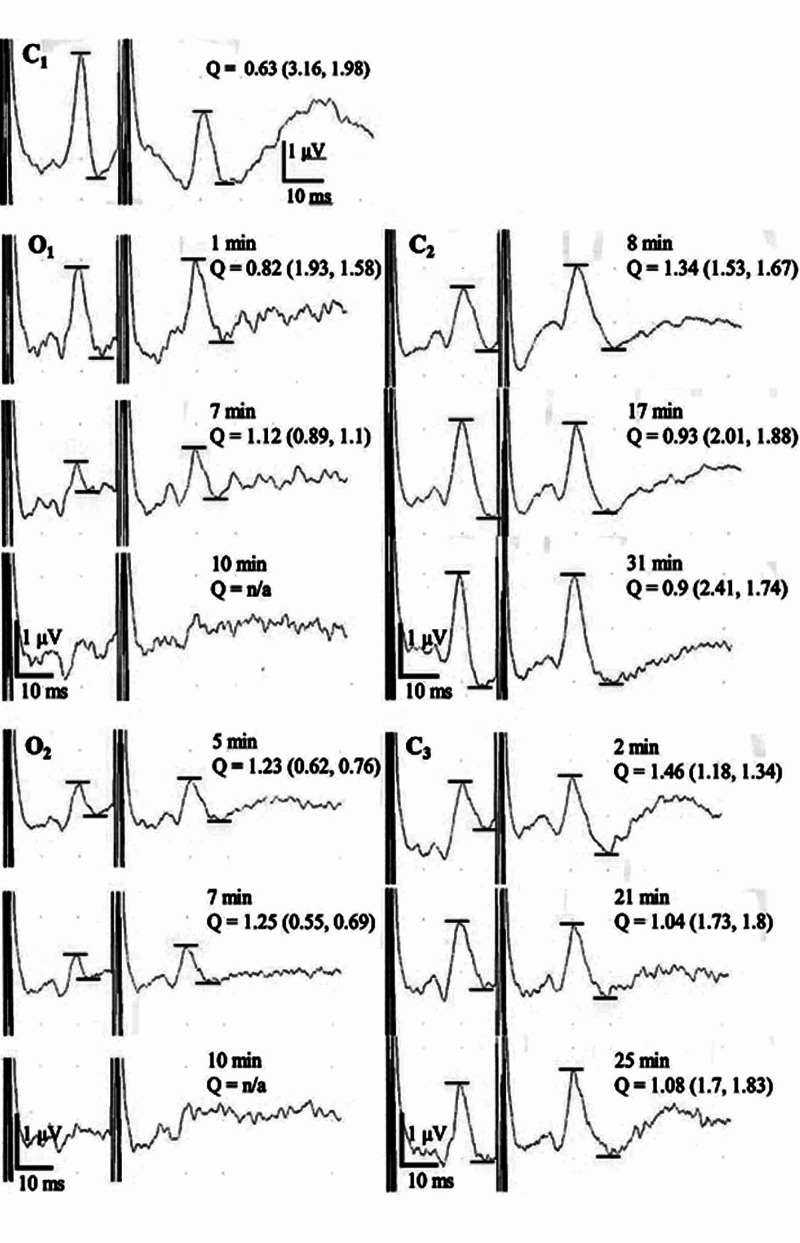
An example of paired-pulse SEPs (No. 21), indicating ischemia-induced neuronal imbalances. C_1_, or controls, indicated paired-pulse suppression (i.e., A_1_ > A_2_, Q = A_2_/A_1_, where Q = 0.63, A_1_ = 3.16, A_2_ = 1.98), with the inter-stimulus interval of 30 ms. The changes in Q values revealed ischemia-dependent neuronal imbalances. In O_1_, occlusion induced a *decrease* in A_1_ from 1.93 (1 min) to 1.12 (7 min), while an *increase* in Q values from 0.82 (1 min) to 1.12 (3 min). At 10 min, both A_1_ and Q values finally abolished due to severe hypoperfusion. In C_2_, release from occlusion reversibly increased A_1_ values from 1.53 (8 min) through 2.01 (17 min) to 2.41 (31 min), while decreased Q values from 1.34 (8 min) through 0.93 (17 min) to 0.9 (31 min). In O_2_, occlusion induced a decrease in A_1_ from 0.62 (5 min) to 0.55 (7 min) and an increase in Q values from 1.23 (5 min) to 1.25 (7 min), the latter of which were larger than those in O_1_, probably due to the increased ischemic load. At 10 min, both A_1_ and Q values abolished again. In C_3_, whereas A_1_ and Q values reappeared soon after release from occlusion (A_1_ = 1.18, Q = 1.46), increased Q values remained almost unchanged (1.04 at 21 min, 1.08 at 25 min), indicating *persistent* *neuronal* *imbalances* (see also No. 21 in Figure 3). The bars indicate peak-to-peak amplitudes. Each number in parenthesis shows amplitudes (uV) of A_1_ and A_2_ respectively.

Paired-pulse SEPs were performed by applying the median nerve stimuli at the wrist contralateral to the affected side, where the peak-to-peak amplitude (measured by N_20_/P_25_ complex) of the second stimuli (A_2_) was divided by that of the first (A_1_; paired-pulse index Q = A_2_/A_1_, see C_1_ in Figure 1). The inter-stimulus interval was 30 ms, wherein paired-pulse suppression (A_1_ > A_2_) was prominent [14].

SEP monitoring was performed using a commercially available device (Neuropack X1, Nihon Kohden, Japan). The overall settings were as follows: filter, 20-1.5 kHz; notch filter, on; recording electrodes, C_3_/C_4_; reference electrode, Fz; stimulation intensity, 20-25 mA; stimulation frequency, 2 Hz; pulse-width, 0.2 ms; and summation, 100-200 times (roughly 1-3 min per single SEP). Since preoperative evaluation of conventional SEPs and A_1_ indicated minimum changes, A_1_ was used as an alternative to conventional SEP. Ischemic warning criteria were set as a >50% decrease in the amplitude of A_1_ [15].

The regional saturation level of oxygen (rSO_2_) of the middle cerebral artery territory was monitored using 2-channel near-infrared spectroscopy (NIRS; INVOS 5100C, Medtronic Inc., Minneapolis, MN, USA), following the focal shaving of the hair over the bilateral fronto-parietal areas. A warning sign was set at a >15% decrease (usually set as >20%) [16].

Data analysis

Off-line analyses of A_1_, Q, and rSO_2_ values were performed for pre-occlusion (C_1_), first occlusion (O_1_), after occlusion with use of shunting (C_2_), second occlusion (O_2_), and after occlusion (C_3_). The data were analyzed by Wilcoxon ranked tests using Bonferroni correction for the multiple datasets, or Mann-Whitney tests for the paired datasets, using the R 3.5.2 software program (https://www.r-project.org/). A p < 0.05 was considered significant. Data were presented as the mean ± standard error (SE) unless otherwise noted.

## Results

Patient characteristics

A total of 34 patients (31 males, three females; mean age: 73.5 ± 1.2 years) were included in the study. The number of patients with hemodynamic deficits of stage 1 and 2 in SPECT was 17 per group (17/34; 50%), respectively. Thirty patients (30/34; 88.2%) had a history of symptomatic ischemia that was associated with neurological deficits over the MCA areas, while four patients (4/34; 11.8%) were asymptomatic. Of these symptomatic patients, persistent or permanent neurological deficits at the time of CEA were confirmed in 19 patients (19/30; 63.3%), all of whom had upper limb deficits to varying extents.

**Figure 2:**
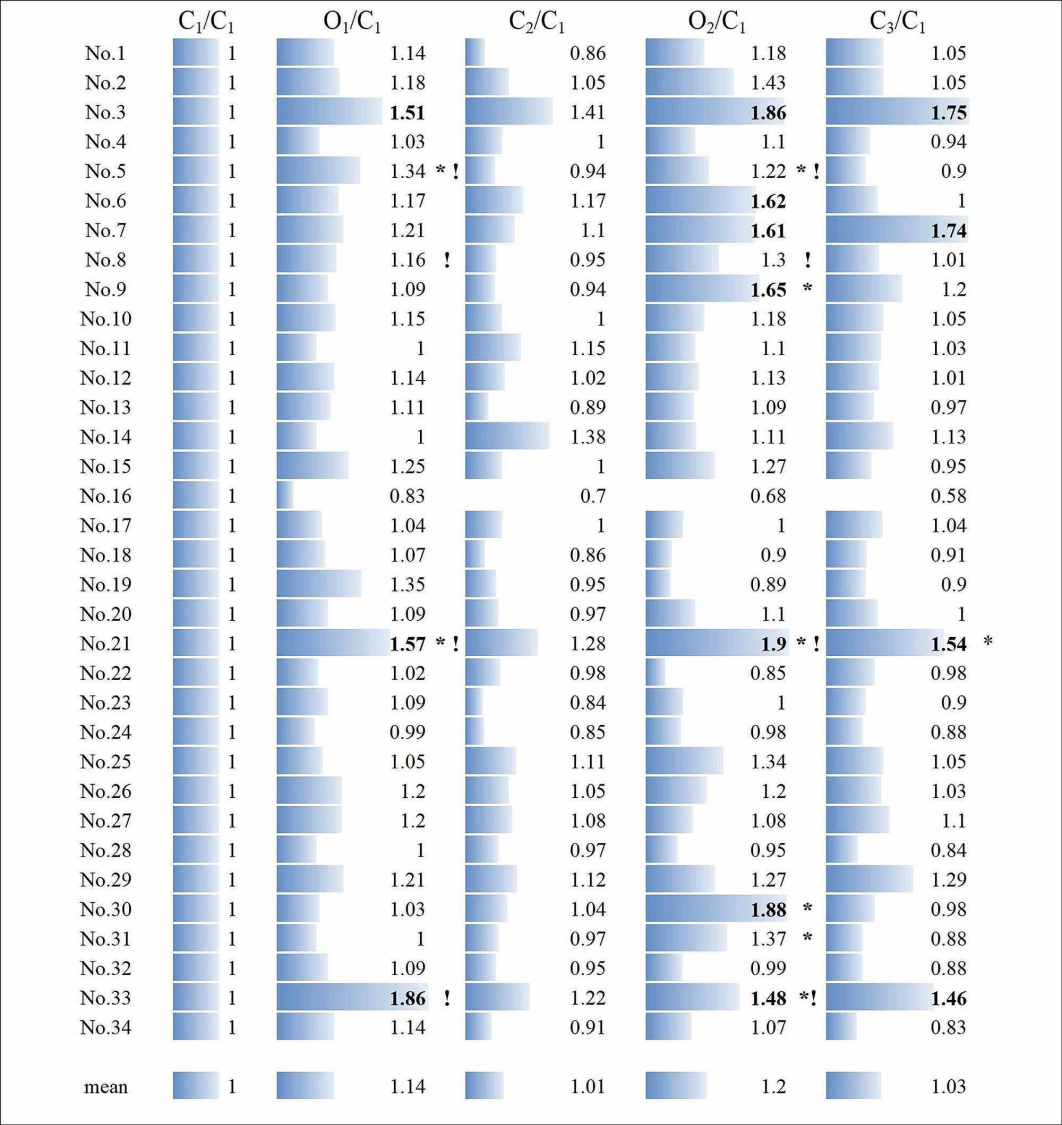
Normalized mean Q values. Normalized mean Q values during C_1_, O_1_, C_2_, O_2_, and C_3_. Persistently increased Q values in four patients (No. 3, No. 7, No. 21, and No. 33) all indicated postoperative DWI abnormalities. Based on the minimum values of 1.46 in No. 33, normalized Q values 46% higher than those in normalized C_1_ were shown in bold. The asterisk (*) and exclamation mark (!) indicate values below criteria detected by A_1_ and rSO_2_, respectively.

While minor postoperative complication was observed in one patient (transient weak motor deficit of the hand in No. 21 [Figure 2]), there were no major post-operative complications such as permanent neurological deficits or hyperperfusion syndrome. The mean carotid occlusion time was 13.7 ± 1.3 min in O_1_ and 17.2 ± 1.7 min in O_2_, with a total mean occlusion time of 30.9 ± 2.2 min.

Intraoperative neuronal imbalances

Mean Dynamics

**Figure 3:**
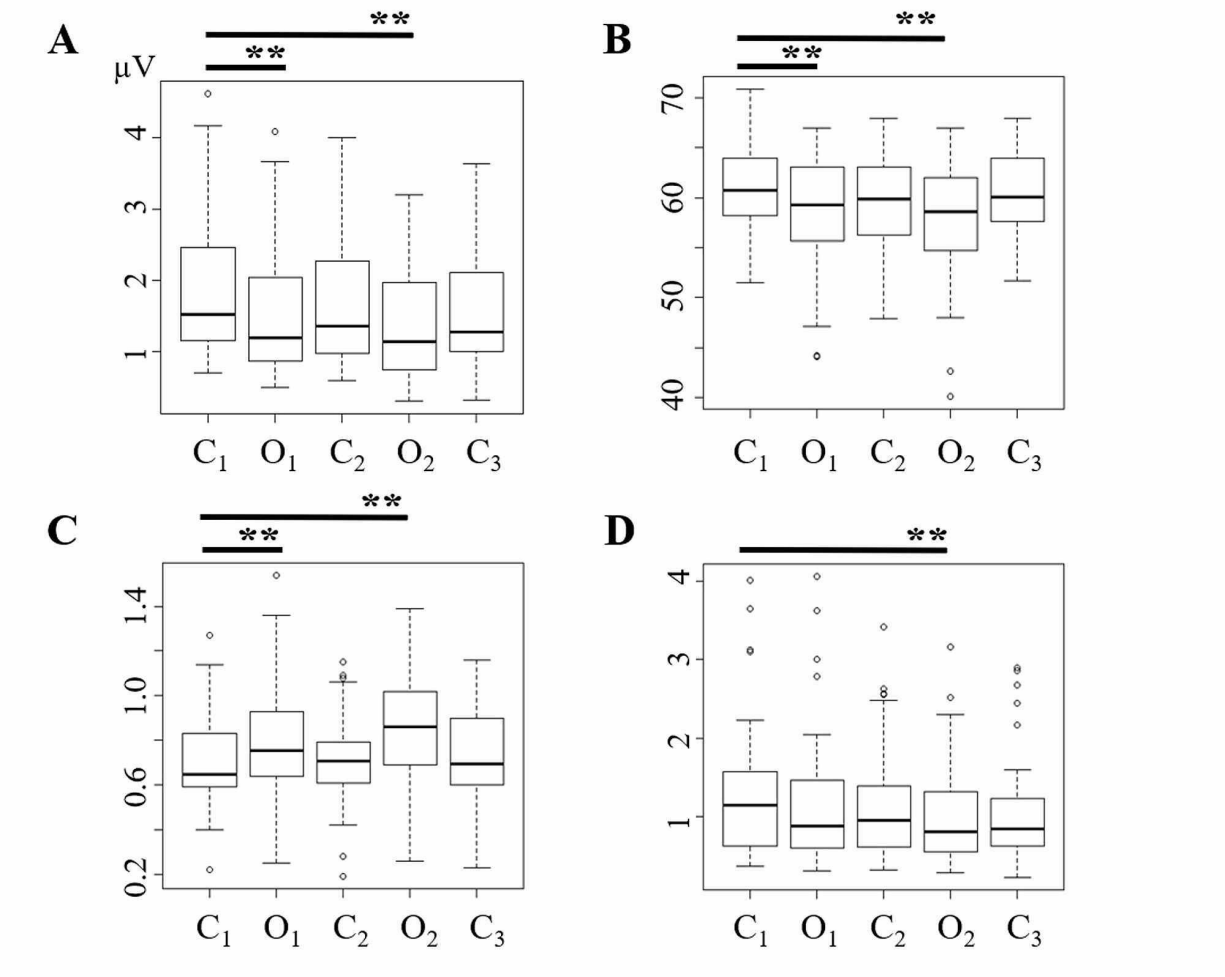
Post-hoc analyses. *Post-hoc* analyses of A_1_ (A), rSO_2_ (B), Q (C) and A_2_ (D). The mean A_1_ values (A) and rSO_2_ values (B) indicated a significant *decrease* during occlusion (O_1_ and O_2_), compared to controls. The mean Q values (C), on the other hand, indicated a significant *increase* during occlusion (O_1_ and O_2_), compared to controls. Whereas mean A_2_ values (D) showed a decreasing trend during occlusion, it became statistically significant only during O_2_, compared to controls. The data were analyzed by Wilcoxon ranked tests using Bonferroni correction for the multiple datasets. The data were shown as the mean ± SE. Statistical significance; p** < 0.001

As expected, carotid occlusion induced a significant *decrease* in the mean values of rSO_2_ (58.36 ± 1.01 during O_1_, p < 0.0001, 57.81 ± 1.04, during O_2_, p < 0.0001), and A_1_ (1.62 ± 0.18 µV during O_1_, p < 0.0001, 1.41 ± 0.15 µV during O_2_, p < 0.0001), compared to their respective controls (60.84 ± 0.79, 2.0 ± 0.19 µV). The levels returned to normal after release from occlusion (59.48 ± 0.87, 1.75 ± 0.17 µV in C_2_, 60.21 ± 0.76, 1.58 ± 0.14 µV in C_3_ [Figure 3A, 3B]).

In contrast, occlusion induced a significant *increase* in mean values of Q (0.81 ± 0.05 during O_1_, p < 0.0001, 0.84 ± 0.04 during O_2_, p = 0.0036) in paired-pulse SEPs, compared with controls (0.7 ± 0.04 [Figure 3C]), which returned to normal levels after occlusion (0.71 ± 0.04 in C_2_, 0.72 ± 0.44 in C_3_). These reversible increases indicated transient neuronal imbalances. Figure 1 shows representative waveforms of paired-pulse SEPs in a case with severe hypoperfusion, wherein an occlusion-induced decrease in A_1_ and an increase in Q values were demonstrated (No. 21, cf. Figure 2).

Compared with those in controls (1.32 ± 1.16), the mean values of A_2_ showed a significant decrease during O_2_ (1.05 ± 0.12, p = 0.003) but not during O_1_ (1.21 ± 0.16, p = 0.11), which returned to normal levels after release from occlusion (1.15 ± 0.13 in C_2_, 1.12 ± 0.12 in C_3_ [Figure 3D]).

Individual Dynamics

**Figure 4:**
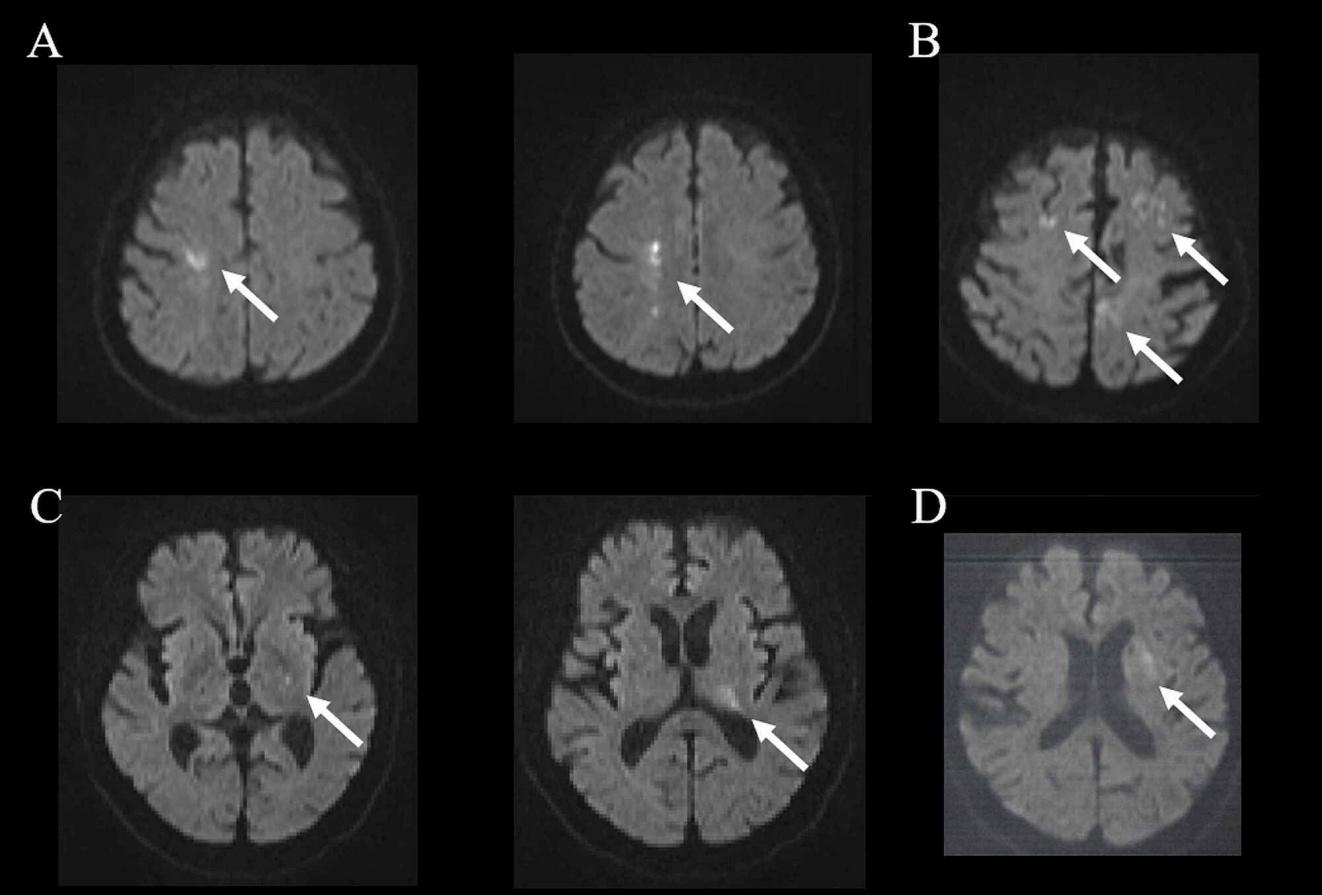
Postoperative DWI abnormalities. Postoperative DWI abnormalities (shown by arrows) in four patients (No. 3 in A, No. 7 in B, No. 21 in C and No. 33 in D).

Warning signs in rSO_2_ were observed in four cases both during O_1_ and O_2_ (4/34; 11.8%, No. 5, No. 8, No. 21 and No. 33, shown by exclamation marks in Figure 2), while those in A_1_ were seen in two cases during O_1_ (2/34; 5.9%, No. 5 and No. 21) and in six cases during O_2_ (6/34; 17.6%, No. 5, No. 9, No. 21, No. 30, No. 31 and No. 33, shown by asterisks in Figure 2). They were all reversible in rSO_2_, but for the one case in A_1_ (1/34; 2.9%, No. 21), having a minor stroke with a transient motor deficit of the hand observed postoperatively for a few days. This patient had transient DWI abnormalities in the contralateral internal capsule and thalamus (Figure 4C).

The increase in Q values after O_2_ was reversible in 30 out of 34 cases (88.2%), without showing any ischemic abnormalities upon DWI MRI (Figure 2). The remaining four cases (4/34; 11.8%, No. 3, No. 7, No. 21 and No. 33), however, exhibited persistent imbalances after release from O_2_, all of which were associated with DWI abnormalities (Figure 4). They were observed over the bilateral parietal cortices (No. 3 and No. 7), the internal capsule, thalamus (No. 21), and the basal ganglia (No. 33) contralateral to the affected side. Q values in these four cases were significantly higher compared with the rest (n = 30) in O_1_ (1.53 ± 0.13 vs. 1.1 ± 0.02, p < 0.0001), O_2_ (1.71 ± 0.1 vs. 1.16 ± 0.04, p = 0.0012) and C_3_ (1.62 ± 0.07 vs. 0.97 ± 0.02, p < 0.0001). These abnormalities were transient, with follow-up MRI performed about one month later not showing any ischemic changes.

**Figure 5:**
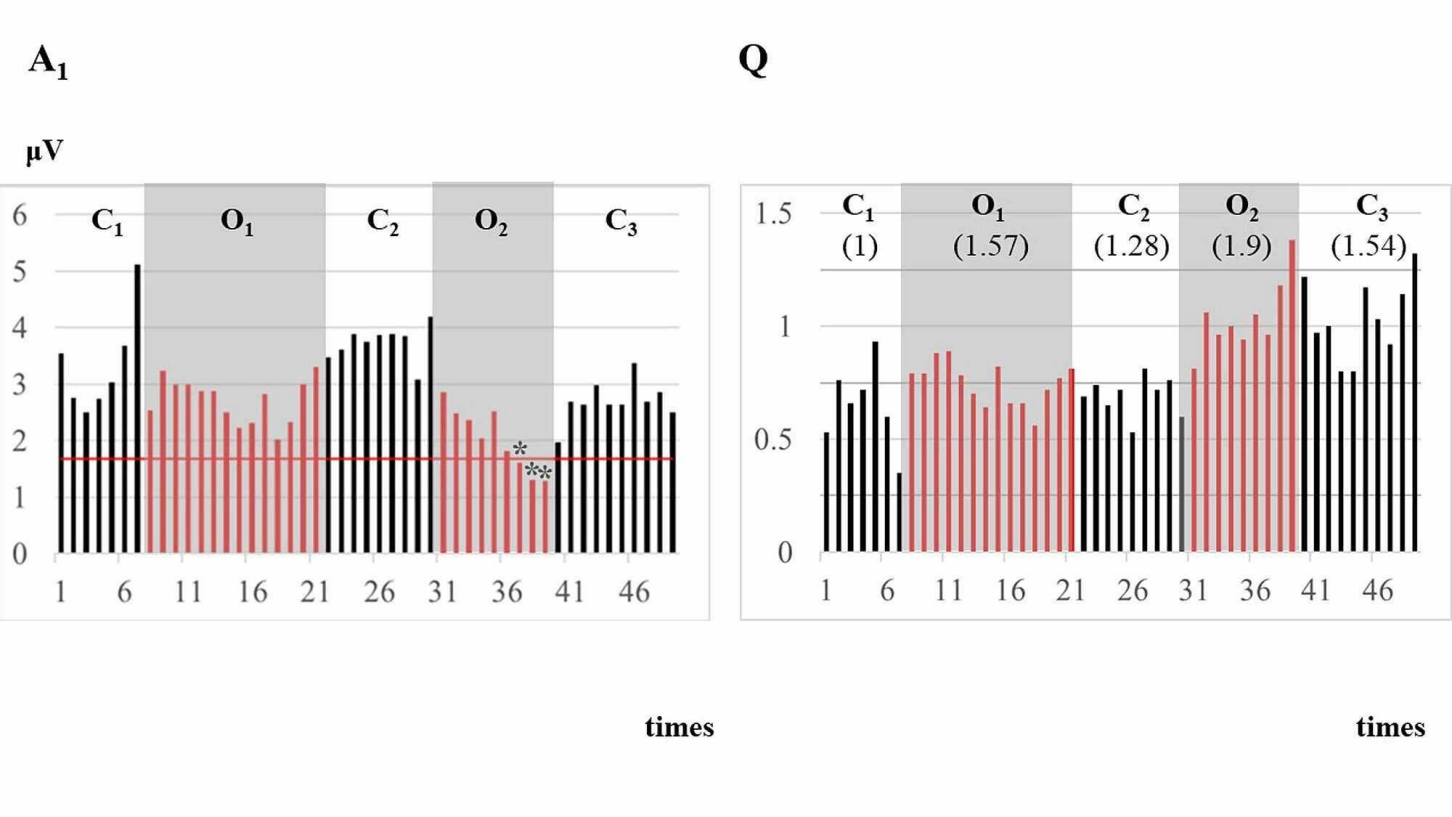
Comparison of A1 (left) and Q values (right) in No. 7. Comparison of A_1_, or conventional single-pulse SEPs, and Q values in a case of No. 7, where postoperative DWI MRI abnormality was identified. This case indicated a false negative in A_1_, while Q values successfully identified ischemic changes through persistent neuronal imbalances. As noted, each occlusion induced a *decrease* in A_1_ but an *increase* in Q values. Whereas A_1_ indicated alarm signs during O_2_ (shown by asterisks), it returned to the normal level (e.g., the values above the ischemic criteria) after occlusion (C_3_), resulting in false negative. Conversely, increased Q values in O_2_ persisted after release from occlusion (C_3_), with the mean value of 1.54 well above a tentative threshold of 1.46 (see also Figure 2), resulting in correct diagnosis. A solid horizontal line and asterisks in A_1_ indicate values below ischemia criteria. Horizontal axis shows the number of trials in SEPs. Each shadow indicates O_1_ and O_2_. Each number in parentheses in the right indicates normalized mean Q values.

Figure 5 showed a representative case of a false negative in A_1_ (No. 7) with a postoperative DWI abnormality. While A_1_ values reached below ischemic criteria during O_2_, they reversibly returned to the normal level in C_3_, leading to a false positive. Q values, on the other hand, indicated persistent imbalances in C_3_.

Preoperative neuronal imbalances

When C_1_ in patients with persistent or permanent neurological deficits over the affected limb area (19/34, 55.9%) were compared with those without (15/34, 44.1%) at the time of CEA, Q values proved to be significantly higher in the former (0.79 ± 0.22 vs. 0.58 ± 0.16, p = 0.006), indicating persistent or permanent neuronal imbalances.

## Discussion

Compared to the investigation of immediate neuronal imbalances after the completion of infarct [17, 18], investigation done before completion of infarct is substantially limited [2, 3]. To our knowledge, this is the first clinical report investigating the real-time changes in neuronal imbalances seen during ischemic monitoring. By complementing false negatives in conventional SEPs [1], paired-pulse SEPs elucidated qualitative criteria for transient, persistent or permanent imbalances, according to the severity of ischemic load. In particular, the association of persistent imbalances with DWI abnormalities implied the clinical availability of ischemic monitoring.

Areas in the penumbra that do not progress to infarction develop SNL, which is rapidly induced (<6 hours from the stroke onset in humans [9-11]), reversible [13], and characterized by a reduction in benzodiazepine receptors in normally-appearing cortex [6, 8]. Animal studies have further shown (i) the induction of SNL in the order of minutes (as early as 5 minutes) through major artery occlusion [5, 19] and (ii) the reversibility of SNL if ischemia is limited to 20 to 60 minutes [10-12]. Given the congruency between SNL and neuronal imbalances in the present study, paired-pulse SEPs may be able to evaluate real-time changes in SNL.

Although the physiological mechanisms underlying paired-pulse SEPs are yet to be elucidated [20], the potential involvement of inhibitory processing has been reported in both basic [21] and clinical studies [20]. A_1_, or the first N_20_-P_25_ component, reflects the overall intracortical activation of the somatosensory cortex. In addition, N_20_ is associated with Brodmann area 3b and the P_25_ component is associated with Brodmann areas 2 and 1, as well as Brodmann area 4 in part [22]. When sensory stimuli were applied at intervals less than 100 ms in humans, paired-pulse suppression (i.e., A_1_ > A_2_) was observed [14]. This suppression is associated with γ aminobutyric acid A (GABA_A_) receptor mediated mechanism [19] through thalamocortical processing [23]. GABA_A_ receptors form GABA_A_/benzodiazepine receptor complex. Thus, given the fact that SNL is associated with the reduction in benzodiazepine receptors, it is physiologically reasonable to assume that changes in GABA_A_-mediated mechanisms can affect the dynamics of Q values. From this view, one simple but plausible explanation for such dynamics would be that, while A_1_ is relatively resistant to pre-penumbral ischemia, GABAergic changes are more susceptible to ischemic changes, which result in neuronal imbalances.

Postoperative DWI abnormalities in the present study were seen in 11.8% (4/34) of the patients, which were roughly in agreement with a meta-analysis study done in CEA patients that demonstrated prevalence of postoperative abnormalities in 10.6% [24]. Whereas this fact potentially indicates a high clinical demand for intraoperative evaluation of DWI abnormalities, the conventional SEPs in the present study indicated low sensitivity. For reference, when DWI abnormalities are targeted, the sensitivity and specificity of conventional SEPs were 25% and 82.4%, respectively. Paired-pulse SEPs reliably detected DWI abnormalities in all cases. There is a possibility, however, that persistent imbalances detected in this study were induced by SNL since SNL is not always associated with DWI abnormalities [25, 26]. This issue must be further investigated using SPECT or positron emission tomography (PET).

Persistent imbalances, if intraoperatively identified, will be managed in the same way as conventional SEPs through intentional hypertension, minimization of time for declamping, and administration of edaravone [27]. Identification of such imbalances will aid in timely and proper postoperative management not only by performing DWI MRI but through prescribing medications, which turned out to be effective for treating impaired cognitive function and inducing functional recovery of the rescued penumbra [7, 8]. Further, paired-pulse SEPs may aid in rapid and proper decision-making for thrombolysis or endovascular therapies.

The limitations of the present study are as follows: (i) the study design, being observational, has inherent limitations; (ii) the small sample size and the limited cases of DWI abnormalities seen in the single institution analyzed in this study may have affected the generalization of the findings; (iii) the physiological mechanisms of Q values and the involvement of SNL were left untouched, with the latter ideally investigated using iomazenil SPECT; and (iv) the paired-pulse SEPs in the study merely presented qualitative criteria of transient, or persistent or permanent imbalances, with the possible existence of ischemic thresholds, as used in Figure 2, still being unknown.

There are still many issues to be addressed, and further research involving large cohorts is needed. Issues of clinical importance will be whether paired-pulse SEPs could be applied to other cerebrovascular surgeries such as aneurysmal clipping, superficial temporal artery to middle cerebral artery (STA-MCA) bypass, or tumor surgeries involving MCA areas. Further, given the increasing opportunities for the perioperative assessment of ischemia due to the development of thrombolysis or endovascular therapies such as thrombectomy, clinical availability of paired-pulse SEPs, if proved useful, will provide rapid and proper decision making with aggressive perioperative management. Finally, it should be clarified whether paired-pulse SEPs can provide a novel diagnostic tool for cognitive impairment in patients with chronic cerebral hypoperfusion.

## Conclusions

Paired-pulse SEPs, in combination with conventional SEPs, may offer better ischemic monitoring. They reliably identified transient, persistent or permanent neuronal imbalances, depending on the ischemic severity, which also suggested that ischemia-induced excitability imbalance may be much more pervasive than previously believed. Taken together, the results are suggestive of potential diagnostic targets for cerebral ischemia.
